# Car Windshield Wiper in the Brain: Case Report

**DOI:** 10.1055/s-0036-1584170

**Published:** 2016-06-10

**Authors:** Zhenpeng Liu, Xianzeng Hou, Xiaoyong Fan, Yuanyuan Hu, Guangcun Liu

**Affiliations:** 1 Shandong University, School of Medicine, Jinan, Shandong, China; 2 Department of Neurosurgery, Qianfoshan Hospital Affiliated to Shandong University, Jinan, China

**Keywords:** penetrating brain injury, transorbital, wiper

## Abstract

**Background** Transorbital intracranial penetrating injury is rare. Damage caused by a huge metallic foreign body is very critical and life-threatening.

**Method** We report an extremely rare case of transorbital intracranial penetrating metal strip (a car windshield wiper), which has not previously been reported in the literature.

**Results** Emergency craniotomy was performed; the object was removed successfully, and the patient's life was saved.

**Conclusion** With the life-threatening penetrating brain injury caused by a huge foreign body, prompt surgical treatment and comprehensive postoperative treatment are important to save patients' lives.

Transorbital intracranial penetrating injury is usually caused by tremendous impact force, which is common in war and occurs sometimes in industrial or traffic accidents. The onset is sudden and results in serious injury. Most injuries are caused by sharp objects such as knives, nails, pencils, chopsticks, and so on. Here, we report an extremely rare case of transorbital intracranial penetrating injury caused by a car windshield wiper, which has never been reported before. In this report, the surgical procedure is reviewed and management of the case is discussed.

## Case Report

A 35-year-old man was injured 11 hours before being admitted to our ward when his car crashed into a roadside ditch. The patient was transported to the hospital by ambulance and was in a deep coma with endotracheal intubation. The Glasgow Coma Scale score was 5/15 points. Just below the left eyebrow, a hard strip of foreign body (car windshield wiper) was observed, with ∼2 cm left outside. The left pupil was 4 mm in diameter and irregular in shape and had no reflex to light; the right pupil diameter was 3.5 mm and oval in shape and had no reflex to light. The limbs of the patient had flexion on the right at pain stimulus.

**Fig. 1:**
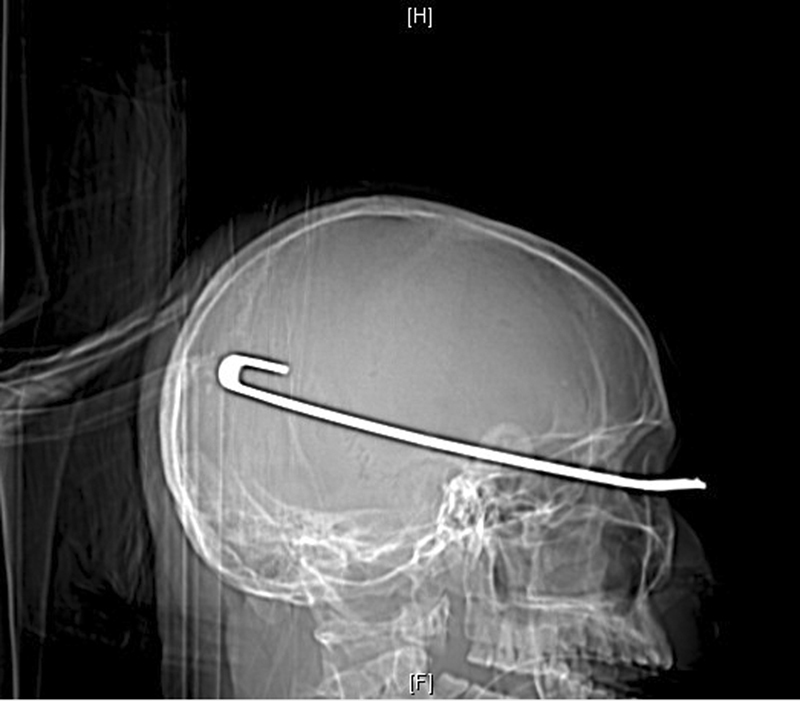
Preoperative brain computed tomography reconstruction showed the wiper with u -shaped rear end.

**Fig. 2:**
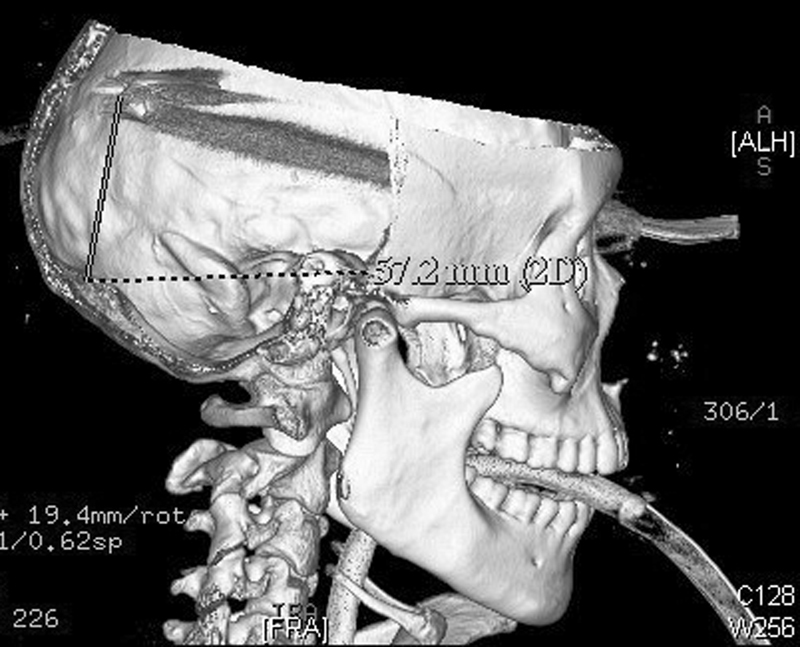
Preoperative skull computed tomography reconstruction.

**Fig. 3:**
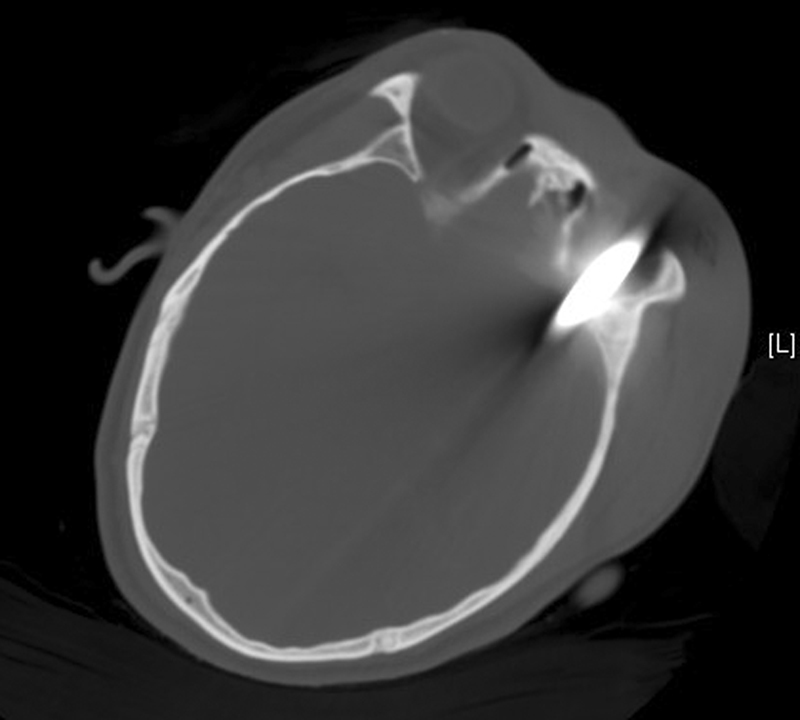
The metal penetrated the cranial cavity through the left orbit.

Urgent head computed tomography (CT) scan plus reconstruction showed the metal strip penetrated the brain through the left superior orbital wall. The foreign body was 18 cm in length, and the end of the metal was u -shaped and located in the left occipital lobe ( Figs. 1 , 2 , and 3 ). A midline shift of the brain was obvious.

The patient's situation was severe and life-threatening, and emergency surgery was necessary. Because the tail of the wiper was like a hook, pulling it out from the front would severely damage the brain tissue. Thus, the surgery was designed to pull the wiper out from the rear. Before the surgery, a temporal-parietal-occipital curved incision with the end of the metal as the center was designed. Meanwhile, an anterior frontal-parietal-temporal incision was reserved in case the incision needed to be expanded. As the wiper penetrated through the sylvian fissure and possibly damaged the sylvian blood vessels, a blood recycling machine was used. With the assistance of the ophthalmologist, the left orbital and intracranial giant foreign body was removed and decompressive craniectomy was performed.

**Fig. 4:**
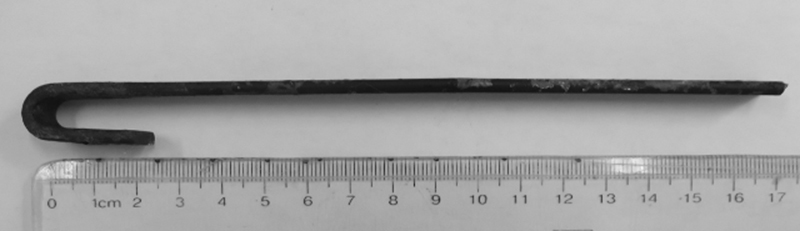
The wiper removed from the patient's brain.

After successful administration of anesthesia, the extracranial part of the metal strip was truncated. After removal of the temporal-parietal-occipital bone flap and dural opening, severe brain swelling and subdural hematoma were observed, and blood gushed from occipital cortex wound. We removed the hematoma and cut the occipital cortex along the wound approximately 2.5 cm until we found the u -shaped rear end of the wiper. The bleeding was severe with exposure of the rear the wiper, so rupture of the sylvian blood vessels was possible. An anterior frontal-parietal-temporal incision was extended forward. A portion of the subdural hematoma was removed and the bottom of the frontal lobe was explored, and we found the wiper penetrating the orbital roof and pulled it out from the rear ( Fig. 4 ). Then we cut the cortex to open the wound tract to find small bleeding spots. When we were ready to end the operation, a massive hemorrhage occurred along the wound tract and the blood pressure dropped to 0. Injury to the left middle cerebral artery M3 was responsible for the massive hemorrhage. We had to coagulate the M3 to stop the bleeding and remove the swelling brain tissue to save the patient's life. The dura was then sutured with reduced tension and an epidural drain was placed. The posterior bone flap was replaced and the front bone flap was removed for decompression. The patient was sent to the Neurosurgical Intensive Care Unit with endotracheal intubation; 8 U red blood cells, 800 mL plasma, and 320 U cryoprecipitate were infused during the surgery.

## Results

**Fig. 5:**
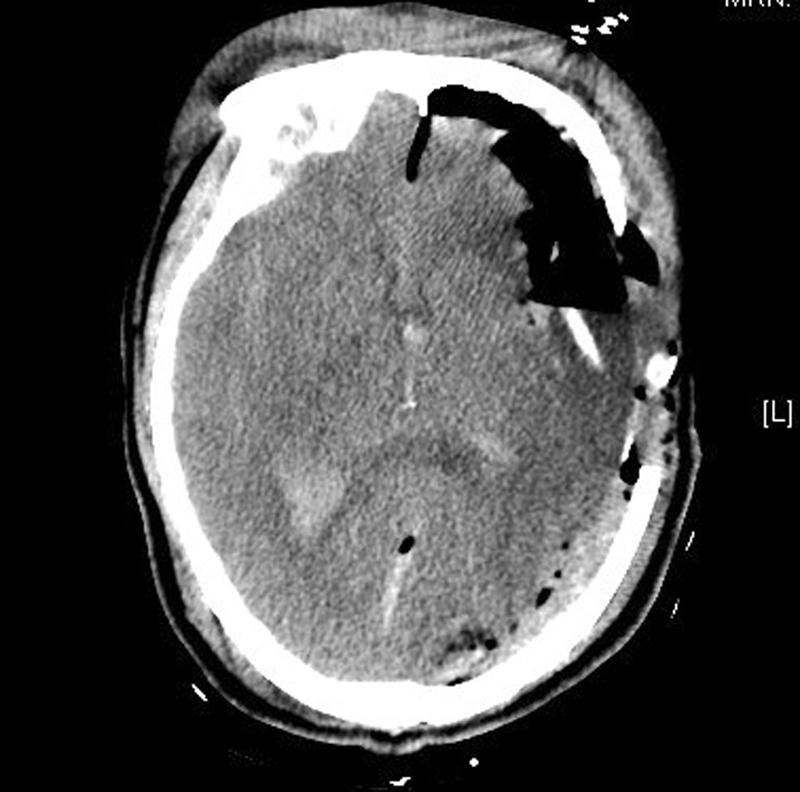
Computed tomography scan on the first day postoperation.

**Fig. 6:**
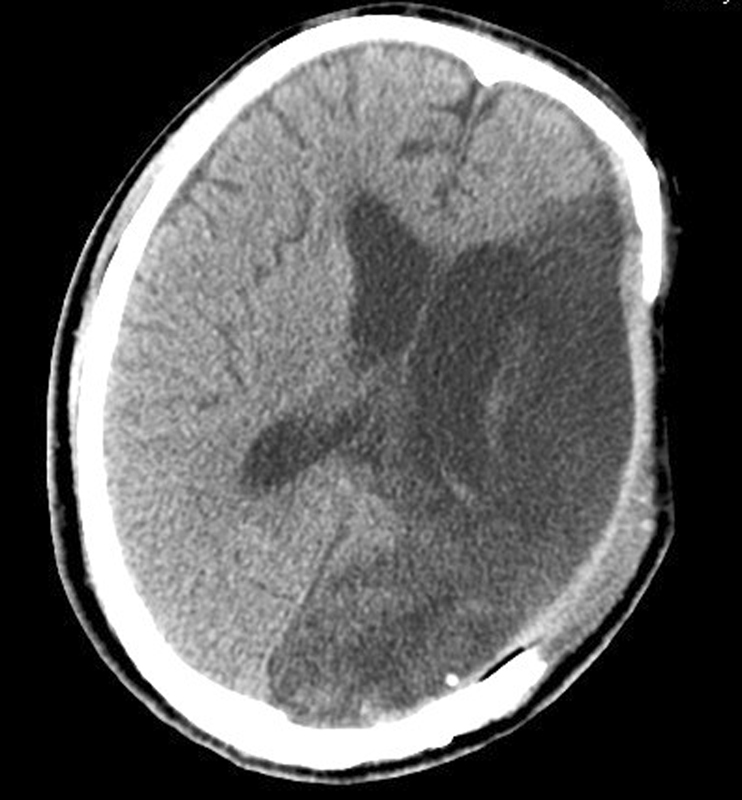
Computed tomography at discharge.

The patient's vital signs were stable after the surgery. The brain CT showed no postoperative hematoma in the surgical cavity, and the midline shift was partially reversed ( Fig. 5 ). The patient underwent tracheotomy on the third day after surgery. Hyperbaric oxygen therapy and acupuncture were implemented for the patient's rehabilitation. The patient was transferred to a local medical center to continue rehabilitation therapy after 75 days in our hospital. At discharge ( Fig. 6 ), the Glasgow Coma Scale score was 9. Both eyes opened unconsciously and had no reflex to light. The left pupil was irregular in shape and fixed, and the right pupil was round but not fixed.

## Discussion

In civilian accidents, most penetrating cranial wounds are caused by knives, nails, spikes, iron rods, pencils, scissors, keys, chopsticks, and screwdrivers. 1 Penetrating head injuries are fatal in 40% of cases because of damage to critical structures, vascular disruption, concussion blast injury, or meningitis. 2 3 In this case, the transorbital intracranial metal object was a car windshield wiper. The intracranial end was u -shaped, and the length was up to 18 cm. The impact force of the traffic accident was tremendous, and we thought that the windshield wiper with u -shaped end directly penetrated into the brain through the superior orbital wall under the eyebrow in this patient. The orbital roof as an entry point is seen in most cases of cerebral penetrating injuries, 5 mainly because when the cranio-orbital penetrating injury occurs, the patient will extend their head backward subconsciously to avoid sharp instrument, exposing the orbital roof wall.

For a high-density object like metal, a CT scan is of significant value in analyzing the size, shape, and location of the object before the surgery. In this case, the brain CT scan and reconstruction was done before the surgery ( Figs. 1 , 2 , and 3 ). The wiper penetrated the frontal-temporal-occipital lobe along the course, and the injuries were serious and life-threatening. The information about the anatomical relationship between the material and vascular system is important. Much can be learned from this case, and the next time, we will perform CT angiography for such an injury.

Saving the patient's life was the priority. In this case, the penetrating injury was complicated by serious bleeding, inflammation, and brain edema and resulted in uncal hernia. Urgent craniotomy was implemented to save the patient's life and to remove the foreign body. However, surgical procedures may aggravate the brain damage, so the relationship between the foreign body and vascular structures should be examined carefully before extraction. 4 For this patient, the fissure vessels were badly damaged by the wiper with severe bleeding. To stop the bleeding, we had to coagulate the middle cerebral artery quickly by bipolar coagulation. A blood recovery device was used, and a large amount of blood product was infused during the procedure.

Cerebrospinal fluid leak, meningitis, and cerebral abscess are the most common postoperative complications for cerebral penetrating injury. 6 The foreign body can cause intracranial infection, and antibiotics are necessary in the therapy. Because long-term confinement to bed can result in a high risk of lung infection, opening an airway and sputum suction were necessary. Previous studies showed that early tracheotomy can shorten the intensive care unit stay and lower the incidence of pneumonia. 7 8 Large doses of antibiotics must be started early and must be based on the report of bacteria cultivation and antibiotic sensitivity. Sputum, cerebral spinal fluid, and secretion of the ocular wound were sent for bacteria cultivation. In a review of transorbital penetrating injuries, early initiation of broad-spectrum antibiotics with good central nervous system availability was recommended. 9 Only when the infection was controlled could the patient progress past the critical stage to lay the foundation for the subsequent rehabilitation. In this case, the patient suffered from high fever with much phlegm. Tracheotomy and continuous lumbar drainage were performed on days 3 and 8, respectively. Multiple sputum culture results were positive for *Klebsiella pneumoniae* and *Staphylococcus aureus* , and reports of cerebrospinal fluid and ocular wound secretions were negative. Thus, according to the results of the sputum culture, antibiotics of β-lactamases, macrolides, quinolones, and vancomycin were combined for treatment until the sputum culture reports were negative and the patient had no clinical presentation of infection. Albumin infusion, intensive care, and nutrition were used to enhance the ability of the body to fight infection. At discharge, the temperature was normal and the condition was stable.

In conclusion, we reported an extremely rare case of transorbital intracranial penetrating injury caused by a car windshield wiper in a traffic accident. The trauma caused extensive and severe damage to the brain because of the huge foreign body and the damage to the fissure vessels. Prompt surgical treatment and comprehensive postoperative treatment with appropriate antibiotics are important in saving patients' lives.
